# Efficacy of Computed Tomography and Ultrasonography in Diagnosis of Metastatic Cervical Lymph Nodes in Orofacial Cancer

**DOI:** 10.22038/ijorl.2021.49018.2628

**Published:** 2021-07

**Authors:** Uche-Albert Okeke, Sunday-Olusegun Ajike, Birch-Dauda Saheeb, Joseph-Bako Igashi

**Affiliations:** 1 *Department of Oral and Maxillofacial Surgery, Ahmadu Bello University Teaching Hospital, Zaria, Kaduna State, Nigeria. *; 2 *Department of Oral and Maxillofacial Surgery, University of Benin Teaching Hospital, Benin-city, Edo State, Nigeria.*; 3 *Department of Radiology, Ahmadu Bello University Teaching Hospital, Zaria, Kaduna State, Nigeria.*

**Keywords:** Computed tomography, Lymph node, Metastases, Orofacial, Sensitivity, Specificity, Ultrasonography

## Abstract

**Introduction:**

There is no consensus on which imaging modality is better for the detection of metastatic cervical lymph nodes in orofacial malignancies. This study evaluates the efficacy of computed tomography (CT) and ultrasonography (US) in diagnosis of metastatic cervical lymph nodes in orofacial cancer.

**Materials and Methods:**

Sixty patients with various histologically diagnosed orofacial malignant lesions and clinical evidence of cervical lymph node metastasis were examined using US and CT. Further, the affected lymph nodes were biopsied and examined histologically. The sensitivity, specificity, positive predictive value (PPV), negative predictive value (NPV), and accuracy of the techniques were calculated. The data was analyzed using Statistical Package for Social Sciences (SPSS) version 19 (SPSS Inc., Chicago, IL, USA) and Microsoft Excel 2010 (Microsoft, Redmond, WA, USA). Test of statistical significance was set at 0.05.

**Results:**

US recorded a sensitivity, specificity, PPV, NPV, and accuracy of 80.0%, 57.1%, 77.5%, 60.0%, and 71.7%, respectively (*P* = 0.004), while CT recorded a sensitivity, specificity, PPV, NPV, and accuracy of 87.1%, 71.4%, 85.0%, 75.0%, and 81.7%, respectively (*P*< 0.0001). Lymph node size was the commonest criterion in the diagnoses of metastases in cases with cervical lymph nodes.

**Conclusion:**

Although we obtained great results using US, our results indicated CT to be a better imaging modality for detecting metastatic cervical lymph nodes in orofacial malignancies.

## Introduction

Accurate evaluation of head and neck malignancies and cervical lymph node status is important for treatment planning and prognosis (1). The presence of metastasis in a cervical lymph node in a patient with oral squamous cell carcinoma decreases the chance of long-term remission by 50 percent when compared with patients who have similar primary tumors without nodal metastasis (2). 

Therefore, the status of cervical lymph nodes is the single most important prognostic factor in oral cancer (3). Although, several studies have assessed the usefulness of ultrasonography (US) and computed tomography (CT) in the diagnosis of metastatic cervical lymph nodes in patients with orofacial malignant lesions, there is no consensus on which of these imaging modalities is superior (3,4). 

US is one of the most widely used imaging technologies in medicine. It is portable, free of radiation risk, and relatively inexpensive when compared with other imaging modalities, such as magnetic resonance imaging (MRI) and CT (5). Different sonographic criteria are available for identification of metastatic cervical lymph nodes. The size criterion varies with the patient population; the most acceptable size criterion selected randomly is 0.9 cm for subdigastric nodes, and 0.8 cm for other cervical nodes, with lymph nodes measuring more than 1 cm considered to be malignant (6). Although larger nodes are more likely to have a higher incidence of malignancy, this criteria can be deceptive because reactive lymph nodes can be as large as malignant nodes (7). Benign nodes tend to be elongated, oval, or elliptical in shape with a long to short axis ratio (L/S ratio) >2.0, whereas malignant lymph nodes tend to have a greater transverse diameter and are more rounded with L/S ratio < 2.0 (8,9). 

Benign nodes are generally homogeneous and hyperechoic, while malignant lymph nodes are typically heterogeneous and hypoechoic. However, none of this information is specific, as malignant nodes may also be hyperechoic and benign lymph nodes may also be hypoechoic (10). 

Van den Brekel and colleagues reported that presence of groups of three or more enlarged nodes at specific drainage sites are predictive of metastases (6). Focal cortical thickening, lymph node necrosis, extracapsular spread, sharp nodal borders, and calcification have also been reported as features peculiar to metastatic lymph nodes as observed through US (6,10-13).

Various CT criteria for assessing cervical lymph metastasis in patients with primary carcinoma of the head and neck have been reported (2,6,14,15). Using the minimal (shortest) axial nodal diameter, nodes exceeding 1.1 cm in the jugulodigastric region and 1 cm in all other cervical nodes are regarded as “probably metastatic” (6). Similarly, spherical nodes, extracapsular nodal spread, nodal grouping, necrosis, calcification, and heterogeneous enhancement on CT are suggestive of metastasis (2,14-17).

There are a limited number of studies that assess the efficiencies of US and CT in the diagnoses of metastatic cervical nodes in patients with orofacial malignancies. To the best of our knowledge, there are currently no studies in peer reviewed journals that compare the efficiencies of US and CT. Therefore, the purpose of this study was to compare the efficacies of US and CT in the diagnoses of cervical lymph nodes metastases in cases with orofacial malignancies.

## Materials and Methods

This prospective comparative study was conducted on both out-patients and in-patients who visited the Oral/Maxillofacial clinic of a regional University Teaching Hospital in Nigeria between July 2014 and December 2015. Sixty consecutive patients who met the inclusion criteria were examined. Sample size was determined using Abramson formula for comparing means (18).

**Inclusion Criteria**

- Patients who gave informed consent.
- Patients with all forms of histologically diagnosed primary malignant orofacial tumors and clinical evidence of cervical lymph node involvement.
- Both male and female patients of all ages

**Exclusion Criteria**

- Patients with evidence of cervical lymph node metastases, with primary lesion not located in the orofacial region
- Patients with cervical lymphadenopathy due to non-malignant orofacial lesions
- Patients with orofacial malignancies without clinical evidence of cervical lymph node involvement
- Patients with orofacial malignant lesions with any co-morbid condition associated with generalized lymphadenopathy.
- Patients with orofacial malignancies and coexisting malignant lesion(s) elsewhere in the body
- Patients who have previously experienced neck dissection.
- Patients who declined to give informed consent.

**Patient examination**

All patients were clinically examined for metastatic cervical lymph nodes, and the site, the size, and the number of such nodes were noted. The site was determined according to level descriptions I-VI given by the Memorial Hospital (Simplified level) Classification of 1981 (19).

Patients were subjected to abdominopelvic US, chest radiographs, and baseline investigations to rule out any concomitant malignant lesion that could metastasize to the cervical lymph nodes. Patients then underwent US of cervical lymph nodes. This was performed with patients in supine position on a couch using Mindray ultrasound system model DC-3 (serial number: mu-0c004357) with highly sensitive probes of 7.5 MHz frequency after application of lubricating (ultrasound transmission), gel on the surface of the area to be scanned. The sonographic criteria used in considering a node positive in this study have been described previously (6,20,21). 

Nodes with sizes greater than 1 cm, nodes that were round in shape with long to short axis ratio (L/S ratio) < 2.0, nodal grouping, hypoechoic structure, presence of cortical thickening, sharp nodal border, calcification, necrosis, and extracapsular spread were considered as positive nodes. CT was performed using a General Electric dual scan hispeed Nx/i CT scanner (serial number: XG0001G-ZR-001. All patients received an intravenous bolus of nonionic contrast medium Iopromide (Ulravist 300; 1.0 mL/kg BW). 

The criteria used in considering a node malignant on CT have been described in previous studies (2,6,14,17). Nodes with size greater than 1 cm, round shape, necrosis, extracapsular spread, contrast enhancement, calcification, high fat density, heterogenous appearance, and nodal grouping were considered as malignant nodes. Suspected metastatic cervical lymph nodes were biopsied, fixed in formalin and sent for histopathology confirmation.

The sensitivity, specificity, predictive values, and accuracy were then calculated as follows:

Sensitivity=True PositiveTrue positive + False negative

Specificity = $\frac{\mathrm{True negative}}{\mathrm{True negative}+\mathrm{False positive}}$

Positive predictive value (PPV) = $\frac{\mathrm{True positive}}{\mathrm{True positive}+\mathrm{False positive}}$

Negative predictive value (NPV) = $\frac{\mathrm{True negative}}{\mathrm{True negative}+\mathrm{False negative}}$

Accuracy = $\frac{\mathrm{True positive}+\mathrm{True negative}}{\mathrm{Total}}$

The collected date was analyzed using Statistical Package for Social Science (SPSS) version 19.0 (SPSS Inc., Chicago, IL, USA) and Microsoft Office Excel 2010 (Microsoft, Redmond, WA, USA). The data was presented in form of tables and figures with statistical significance tested using Pearson Chi-square test (χ^2^) and set at 0.05. 

Approval for this study was obtained from the Health Research Ethics Committee of the institution (reference number: ABUTH/ HREC/ J03/2013). Patient participation was entirely voluntary with ensured confidentiality.

## Results

Sixty patients with orofacial malignant lesions and suspected cervical lymph node metastasis were recruited in this study. There were 37 (61.7%) males and 23 (38.3%) females giving a male to female ratio of 1.6:1. 

The ages ranged from 9 to 75 years, with a mean age of 49.3 ± 1.58 years and a peak age incidence in the 7th decade (Table 1). Association between age and incidence of orofacial malignant lesion was statistically significant (*P *= 0.024). 

**Table 1:** Age and sex distribution of 60 patients with orofacial malignant lesions and clinical evidence of cervical lymph node metastasis

Age group (years)	Male		Female		Total	
	n %		n %		n %	
0-9	-	-	1	(1.7)	1	(1.7)
10-19	-	-	1	(1.7)	1	(1.7)
20-29	3	(5)	3	(5)	6	(10)
30-39	8	(13.3)	3	(5)	11	(18.3)
40-49	5	(8.3)	3	(5)	8	(13.3)
50-59	7	(11.7)	5	(8.3)	12	(20)
60-69	7	(11.7)	6	(10)	13	(21.7)
70-79	7	(11.7)	1	(1.7)	8	(13.3)
** Total** **P = 0.024**	37 (61.7)		23 (38.4)		60 (100)	
			

**Fig 1:**
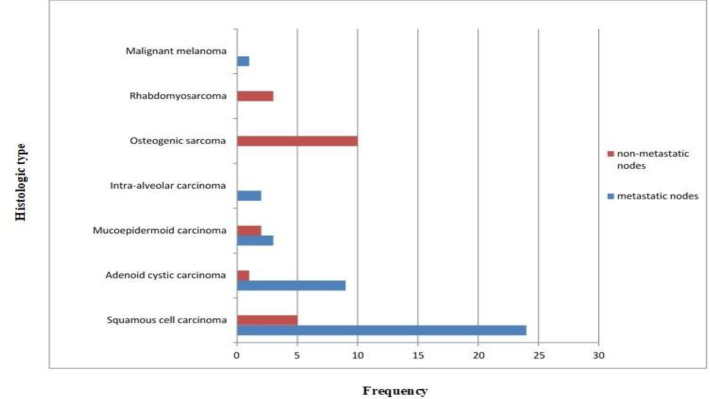
Relationship between tumor histology and cervical lymph node metastasis

The relationship between tumor histology and lymph node metastasis revealed that, while 24 patients with squamous cell carcinomas had lymph node metastasis, none of the sarcoma patients exhibited lymph node metastasis (Figure 1).

Of the 60 patients, 39 (65%) patients were confirmed histologically to possess positive cervical lymph node metastasis, while 21 (35%) patients were negative (Table 2). US recorded 31, 9, 12, and 8 patients as true positives, false positives, true negatives, and false negatives, respectively (Table.2), indicating a sensitivity, specificity, PPV, NPV, and accuracy of 80.0%, 57.1%, 77.5%, 60.0%, and 71.7%, respectively (p-value = 0.004) (Table.3). CT, on the other hand, recorded 34, 6, 15, and 5 patients as true positives, false positives, true negatives, and false negatives, respectively (Table 2), indicating a sensitivity, specificity, PPV, NPV, and accuracy of 87.1%, 71.4%, 85.0%, 75.0%, 81.7%, respectively (p-value < 0.0001) (Table.3).

**Table 2:** Comparison of US, CT, and Histological Examination

Ultrasonography	**Positive**	**True positive (n = 31)**	**False positive (n = 9)**
	Negative	False negative (n = 8)	True negative (n = 12)
**Total **	**n = 39**	**n = 21**	
Investigation	Findings	Histopathological Positive	Examination Negative
**Computed**	Positive	True positive (n = 34)	False positive (n = 6)
Tomography	Negative	False negative (n = 5)	True negative (n = 15)
** Total**	**n = 39**	**n = 21**	
			
			

**Table 3:** Sensitivity, specificity, positive predictive value (PPV), negative predictive value (NPV), accuracy, and p-values of US and CT

Statistical Parameter	Ultrasonography (%)	Computed tomography (%)
**Sensitivity **	**80.0**	**87.1**
**Specificity **	**57.1**	**71.4**
Positive predictive value	77.5	85.0
Negative predictive Value	60.0	75.0
Accuracy	71.7	81.7
Pearson Chi-square test (χ^2^)	P= 0.004	P < 0.0001
		

**For both US and **CT**, the highest sensitivity of 97.4% was recorded with respect to nodal size. Only one case (sensitivity: 2.6%) and two cases (sensitivity: 5.1%) of nodal calcification were documented for US and **CT, **respectively (**Tables 4** and** 5, respectively).

**Table 4:** US criteria for diagnosis of metastatic cervical lymph nodes

Criteria	True	False	True	False	Sensitivity	Specificity	Positive	Negative	Accuracy
	**Positive** **(n)**	**Positive (n)**	**Negative (n)**	**Negative (n)**	**(%)**	**(%)**	**Predictive** **Value (%)**	**Predictive** **Value (%)**	**(%)**
**Size** **(>1 cm)**	38	8	13	1	97.4	61.9	82.6	92.9	85.0
**Shape** **(Rounded)**	30	12	9	9	76.9	42.9	71.4	42.9	65.0
**Echogenicity** **(Hypoechoic)**	27	8	13	12	69.2	61.9	77.1	61.9	66.7
**Grouping**	11	5	16	28	28.2	76.2	68.8	76.2	45.0
**Cortical** **Thickening**	13	6	15	26	33.3	71.4	68.4	71.4	46.7
**Nodal** **Calcification**	1	1	20	38	2.6	95.2	50.0	34.5	35.0
**Necrosis**	19	13	8	20	48.7	38.1	59.4	28.4	45.0
**Sharp Nodal** **Border**	29	12	9	10	74.4	42.9	70.7	42.9	63.3
**Extracapsular** **Spread**	18	11	10	21	46.2	47.6	62.1	32.3	46.7
									

**Table 5:** CT criteria for diagnosis of metastatic cervical lymph nodes

**Criteria**	**True**	**False**	**True**	**False**	**Sensitivity**	**Specificity**	**Positive**	**Negative**	**Accuracy**
	Positive (n)	Positive (n)	Negative (n)	Negative (n)	(%)	(%)	Predictive Value (%)	Predictive Value (%)	(%)
Size (>1 cm)	38	7	14	1	97.4	66.7	84.4	93.3	86.7
Shape (Rounded)	33	9	12	6	84.6	57.1	78.6	66.7	75.0
Grouping	11	6	15	28	28.2	71.4	64.7	34.9	43.3
Calcification	2	1	20	37	5.1	95.2	66.7	35.1	36.7
Extracapsular Spread	23	12	9	16	58.9	42.9	65.7	36.0	53.3
Necrosis	24	12	9	15	61.5	42.9	66.7	37.5	55.0
Fat density	6	5	16	33	15.4	76.2	54.5	32.7	36.7
Contrast Enhancement	26	11	10	13	66.7	47.6	70.3	43.5	60.0
Heterogenous Appearance	23	8	13	16	58.9	61.9	74.2	44.8	60.0
									

## Discussion

The histologically positive nodes of 38 carcinoma patients in this study were metastatic, while all nodes found in sarcoma patients were confirmed to be negative on histology. These findings were in consonance with previous studies, which reported that carcinomas spread through lymphatics while sarcomas are mainly hematogenous (22, 23). Lymph nodes of patients with sarcomas were reported as histologically reactive hyperplasia attributable to secondary infection of the tumors. There was one case of malignant melanoma with a positive node, which was in agreement with the findings of Dadras et al. (24) that malignant melanomas, though rare, are highly metastatic lesions with high recurrence rate.

The sensitivity of US in this study was similar to that reported in previous studies (4,6,25). However, this sensitivity was high when compared with the sensitivity of 47% - 68.7% reported in some other reports (3,26). The high sensitivity in this study might be attributed to differences in scanning techniques employed by the different radiologists when compared with similar studies that reported lower sensitivity. The radiologist in this study focused on single cervical node per patient, thereby reducing fatigue and bias, compared to previous studies that involved scanning of multiple cervical nodes and reported lower sensitivity (26). Secondly, patients in this study exhibited clinically palpable suspected cervical lymph node metastases, unlike a similar study that examined only the patients with no clinically palpable cervical nodes (N0) (3). 

The 87.1% sensitivity for CT in this study was in accordance with the findings of previous studies, which reported sensitivities ranging from 77.5% to 95.6% (4, 27, 28), but higher than the findings of some other studies, which reported sensitivity of 50% to 61% (29-32). However, the sensitivity reported in this study was lower than the sensitivity of 100% reported by Mancuso et al. (33). 

The high sensitivity of CT reported in this study can be attributed to the large size of the nodes examined, the experience of the radiologist, the use of nine criteria in assessing metastatic cervical nodes, and the use of a non-ionic contrast medium Iopromide (Ulravist). Most metastatic cervical nodes showed contrast enhancement similar to the primary tumour, thus supporting the previous report by Noworolski and colleagues (17). 

There is still no consensus on which one of the two imaging techniques is superior in the detection of metastatic cervical nodes in cases with orofacial malignant lesions. Although, some studies reported US to be better than CT with better sensitivity, specificity, and accuracy (4,34), the findings in this study reported CT to be better than US on the basis of sensitivity, specificity, accuracy, PPV, and NPV.

The increased sensitivity of US and CT in this study may be attributed to late presentation by most patients due to ignorance, poverty, low index of suspicion, and delayed referrals by medical personnel in rural areas. At presentation, most patients had large palpable cervical nodes with many patients exhibiting features of metastasis using both imaging techniques. The commonest criterion in this series using US was the size of the lymph nodes. Lymph nodes measuring more than 1 cm were considered to be metastatic in this study, which reaffirmed the findings of Dayanand et al. (25). 

Nodal size was also recorded as the commonest CT criterion for the diagnosis of metastatic cervical lymph nodes. This was similar to the findings of another study, which reported that the most widely accepted parameter for differentiating between normal and metastatic nodes on CT was the size of the lymph nodes (35). However, central necrosis was documented as the commonest criterion in another study (6). 

*Limitation of the study*

Interpretation of US and CT results were dependent on the expertise and experience of the radiologist. 

## Conclusion

Though US was very useful in the diagnosis of cervical lymph node metastasis, CT recorded a higher sensitivity and better accuracy, and thus, was considered to be a better imaging modality for the detection of metastatic cervical nodes in orofacial malignancies. However, histopathological examination remains the gold standard for the diagnosis of cervical lymph node metastasis.
